# Can anthropometric, body composition, and bone variables be considered risk factors for musculoskeletal injuries in Brazilian military students?

**DOI:** 10.1186/s12891-018-2292-3

**Published:** 2018-10-17

**Authors:** Mauro A. S. Melloni, Josiel De Almeida Ávila, Mauro Alexandre Páscoa, Camila Justino De Oliveira Barbeta, Vagner Xavier Cirolini, Ezequiel M. Gonçalves, Gil Guerra-Júnior

**Affiliations:** Departamento de Pediatria, Universidade Estadual de Campinas-Unicamp, Tessália Vieira de Camargo, 126, Cidade Universitária Zeferino Vaz, Campinas, SP Zip code: 13083-887 Brazil

**Keywords:** Military, cumulative trauma disorders, body composition, risk factors

## Abstract

**Background:**

Musculoskeletal injuries are the main cause of premature discharge from military service and can sometimes lead to permanent disabilities. Some intrinsic risk factors are well discussed in the literature. However, the relation between body composition variables and the risk for musculoskeletal injury is not well known or recognized.

**Methods:**

This prospective study evaluated 205 Brazilian military students. At the beginning of military service, health status and sports experience prior to military service were registered. Anthropometric variables were evaluated, and bone and body composition variables were measured using dual-energy X-ray absorptiometry. The occurrence of musculoskeletal injuries throughout the year was registered at the military physiotherapy service. At the end of 1 year of follow-up, risk factors were analysed by comparing the variables between the injured and non-injured students.

**Results:**

No difference in previous health status was found between injured and non-injured groups, whereas sports experience prior to military service was identified as a protective factor (Odds Ratio (OR) 0.323; 95% CI: 0.108–0.968; *p* = 0.044). Anthropometric, bone, and body composition variables could not be identified as risk factors for musculoskeletal injuries in Brazilian military students.

**Conclusion:**

Anthropometric, bone, and body composition variables could not be considered risk factors for musculoskeletal injuries in Brazilian military students.

## Background

Being healthy and physically fit is required in the military profession. Thus, some aspects of military physical training programs are important to ensure development of the physical and fitness skills required in the military profession. However, they can also lead to musculoskeletal injury (MI) and disabilities, which in turn result in premature discharge from military service [1, 2]. In this respect, a systematic review [2] showed a cumulative incidence ranging from 8 to 51% for MI related to military physical training, whereas a prospective study reported that almost 70% of participants followed up for 6 months presented with at least one type of MI, [3] concluding that MI is an important public health problem for the military.

The literature on general military health usually recognizes that overload injuries are more prevalent than traumatic injuries in the military population [4]. Furthermore, MI is highlighted as the main cause of premature discharge from military service [5] and the main reason for seeking medical care during the service [1]. Consequently, premature discharge from military service and the need for medical care owing to MI can result in financial and physical fitness losses and psychological changes, mainly in countries where military service is compulsory [5].

Many variables are reported as risk factors for MI related to military physical training. In general, these variables are usually classified as extrinsic (e.g. long weekly running distance, absence of sports experience prior to military service, smoking habit, and history of MI prior to military service) [5, 6] or intrinsic (e.g. low physical fitness, low educational level, large abdominal girth, high body mass index [BMI], and low body mass) [3–5].

Previous military studies have sought to identify anthropometric characteristics as risk factors for MI related to physical training. Those studies observed that BMI [3–5, 7] and waist circumference [3] were potential risk factors for MI. Thus, as the literature has selected anthropometric variables as risk factors, we hypothesized that a more specific body composition assessment can provide information regarding the predictive value of body components as risk factors for injuries in militaries.

In this regard, a Greek study that measured body fat percentage using bioelectrical impedance analysis confirmed the hypothesis by observing that adiposity expressed as body fat percentage can predict the risk for MI in militaries [8]. However, a glance at the literature exposes the current gap on this topic, because there are few studies that have investigated body composition variables as risk factors for MI. For example, to our knowledge no recent studies have measured fat-free mass, bone mineral content (BMC), and bone mineral density (BMD) to investigate the risk factors for MI in militaries. In this respect, dual-energy X-ray absorptiometry (DXA) is a well-adopted method to evaluate body composition in different populations, including children and adolescents, [9–11] individuals with different diseases [12] athletes, [13, 14] and also militaries, [15] mainly because it is considered non-invasive and fast and involves low radiation exposure [16–18]. Furthermore, another advantage of this method is that it can evaluate total or segmental (right and left sides of the upper and lower limbs and trunk) fat mass, BMC, and lean soft tissue as separate compartments with good accuracy and reliability [16–18], which can provide information regarding imbalances among different body tissues and segments. These imbalances can supposedly represent an additional risk for MI, as observed by a study on rugby athletes [19]. In that study, lower BMD, lower fat-free mass, higher fat mass, and higher body mass were considered risk factors for bone injuries [19]. Furthermore, considering that comparative tests have identified imbalances in limb performance as risk factors for MI [20, 21], we believe that it is important to investigate if there are relationships between body composition imbalance in terms of limbs and the risk of injury. To our knowledge, this has not been investigated, especially using DXA. On the other hand, despite several advantages and the accuracy of DXA, its high cost and the need to visit specific research centres for evaluation may explain why few studies have adopted this method for investigating the risk factors for MI.

Thus, considering the fact that there are many studies focusing on the investigation of anthropometric variables as risk factors for MI in different populations, no studies have used DXA in militaries for the investigation of body composition as a risk factor for MI, this study aimed to verify the prevalence of MI in military students and to investigate the effect of total and segmental body composition assessed by DXA on the risk for MI in military students at the end of 1 year of military service.

## Methods

This was a prospective study with a follow-up period of 9 months (from March to November 2013). The participants were military students from Escola Preparatória de Cadetes do Exército located in Campinas, São Paulo, Brazil. This school is responsible for the first year of study of cadets in the Brazilian army and annually receives 500 students approved in a public contest under a boarding school regime. The first 205 male students who agreed to participate in this study were included in a convenience sample. The inclusion criteria were recent inclusion in the army during the study period and the absence of any physical complaint or MI at the baseline evaluation at the beginning of military service. The study was approved by the ethics committee of the faculty of medical science of the Unicamp (n° 511.4610).

### Baseline measurements

Participants underwent health status and body composition evaluation at the beginning of service in March 2013. Prior evaluation was performed at the Laboratory of Growth and Development in the Pediatric Investigation Center, University of Campinas, Campinas, São Paulo, Brazil. Participants were required to fill out a questionnaire that assessed demographic data and other potential risk factors such as history of chronic disease and MI prior to military service and physical activity experience.

Body mass was measured using a balance-beam scale (Filizola™) with a precision of 100 g that was graduated from 0 to 150 kg. Height was measured using a stadiometer (Holtain Ltd.™) with a precision of 1 cm. BMI was calculated using the formula weight/height^2^.

### Body composition and bone variables

Body composition and bone variables were measured using DXA (model iDXA, GE Healthcare Lunar, Madison, WI, USA). Fat mass (kg) and relative fat mass (%), BMC (kg), fat-free mass (kg), and BMD (g/cm^2^) were estimated. In order to maximize the investigation on risk factors, we analysed the data for total and segmental (right and left sides of the lower and upper limbs and trunk) body composition and bone variables.

### Training routine and injury registration

**Fig. 1:**
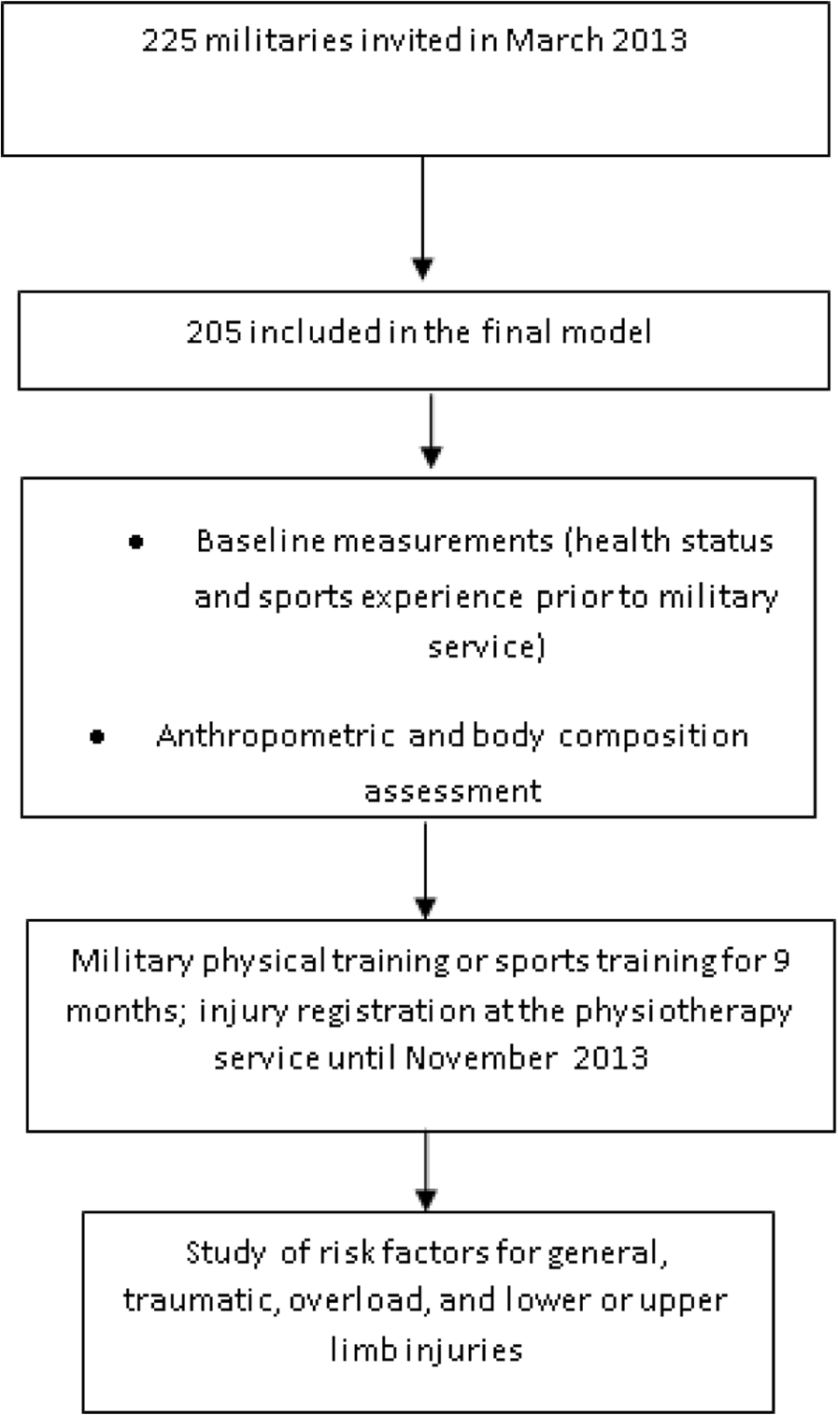
Study sequence actions. Legend: flow of participants through the study

After baseline evaluation, participants started the military physical training program proposed and coordinated by the military school. This program comprised five weekly training sessions that each lasted for 1 h and 30 min. After some training sessions, according to their sporting abilities, some participants were selected to form school sports teams that would represent the military school in Brazilian military competitions. Consequently, a specific training period during the year was planned for some students according to the sports modality that they were recruited for. Sports training and military physical training were conducted at the same period. In case of health complaints, participants sought military school medical service. MI was diagnosed by a military physician and was defined as a musculoskeletal complaint that led to at least one instance of withdrawal from training or competition. In this case, the participant was referred to a military physiotherapy service, and the researcher proceeded with the injury registration. Injuries were classified according to aetiology as traumatic (a known trauma in a specific moment) or overload (non-traumatic mechanisms). At the end of the study period, we proceeded with the investigation on risk factors for general, traumatic, and overload injuries and lower and upper limb injuries (Fig. 1).

### Statistical analysis

Data were analysed using SPSS version 16. Kolmogorov–Smirnov test was used to verify the normality of data. Independent-samples Student’s t-test was used to compare the injured and non-injured militaries. To verify the association between MI and sample characteristics (sports team participation during the study, history of chronic disease or MI prior to military service, and sports experience prior to military service), chi-square test or Fisher’s exact test was used. Logistic regression analysis was used to investigate the risk factors for MI, traumatic or overload injuries, and upper or lower limb injuries. The computation of odds ratios was included. The significance level was set at 5%.

## Results

During follow-up, 66 injuries were registered in 56 participants (27.3% of the sample), with 41 overload injuries (62.1%) and 25 traumatic injuries (37.8%). The most prevalent injuries observed were ankle sprain (16.6%) and medial tibial stress syndrome (15.1%). Most injuries occurred in the lower limbs (69.7%), followed by the upper limbs (25.7%) and spine (4.5%). The demographic and descriptive data of the total sample and comparison of anthropometric and body composition variables between the injured and non-injured groups are presented in Table 1. No differences between the injured and non-injured groups were found.

**Table 1:** Demographic data of the total sample and comparison of anthropometric and body composition variables between the injured and non-injured groups

	Total *n* = 205				Non-injured *n* = 149		Injured *n* = 56		*P*
Variables	Mean	SD	Min	Max	Mean	SD	Mean	SD	
Age (years)	19.6	1.4	16.7	23.9	19.5	1.5	19.8	1.4	0.168
Height (cm)	176.2	6.4	160.3	192.4	175.8	6.4	177.3	6.1	0.111
Body mass (kg)	71.3	8.1	53.8	95.5	71.3	8.5	71.2	7.0	0.889
BMI (kg/m^2^)	22.9	2.1	17.62	28.8	23.1	2.1	22.6	2.0	0.187
FM (kg)	12.5	3.4	5.7	22.3	12.5	3.4	12.4	3.6	0.914
%FM	17.2	3.7	9.7	26.1	17.2	3.5	17.2	4.1	0.979
FFM (kg)	56.3	5.9	41.3	75.4	56.3	6.2	56.1	5.3	0.856
BMC (kg)	3.0	0.4	2.2	4.2	3.0	0.4	3.0	0.4	0.916
BMD (g/cm^2^)	1.229	0.095	0.989	1.561	1.230	0.093	1.227	0.101	0.874

*Abbreviations: SD* standard deviation, *BMI* body mass index, *FM* fat mass, *%FM* relative fat mass, *FFM* fat-free mass, *BMC* bone mineral content, *BMD* bone mineral density

Similarly, no differences in sports team participation during the study (Chi-Square 0,010; *p* = 0.92), history of chronic disease (Fischer exact *p* = 0.913) or MI (Chi-Square = 2593; *p* = 0.107) prior to military service, and sports experience prior to military service (Fisher exact *p* = 0.51) were found between the injured and non-injured groups.

Table 2 shows the results of logistic regression analysis to investigate the included variables as risk factors for MI.

**Table 2:** Logistic regression analysis of risk factors with respect to general sample characteristics for categorical variables

Variables	B	SE	OR	95% CI OR	*P*
Non-athlete^a^	0.078	0.312	1.081	0.587–1.994	0.802
No disease^b^	0.041	0.613	1.042	0.313–3.465	0.947
No previous injury^c^	0.553	0.316	1.739	0.935–3.233	0.080
Sports practice^d^	−1.129	0.559	0.323	0.108–0.968	0.044

*Abbreviations: B* beta coefficient of logistic regression, *SE* standard error, *OR* odds ratio, *95% CI* 95% confidence interval, *BMI* body mass index

^a^non-athlete during the follow-up

^b^no history of chronic disease prior to the service

^c^no history MI prior to the service

^d^sports experience prior to military service

Anthropometric variables were not found to be risk factors for MI (height OR = 1.040, 95% CI = 0.991–1.093, *p* = 0.112; body mass OR = 0.997, 95% CI = 0.960–1.036, *p* = 0.888; BMI OR = 0.903, 95% CI = 0.776–1.051, *p* = 0.187). An absence of sports team participation during follow-up and an absence of history of chronic disease or MI prior to military service were not found to be protective factors for MI. In contrast, sports experience prior to military service was found to be a protective factor for MI (OR = 0.32; 95% CI 0.108–0.968; *p* = 0.04). Logistic regression analysis for categorical variables are shown in Table 2.

The results of logistic regression analysis to investigate body composition variables as potential risk factors for general, traumatic, and overload injuries are presented in Table 3. None of the studied variables were found to be risk factors.

**Table 3:** Logistic regression analysis to investigate body composition variables as risk factors for general, traumatic, and overload injuries

		General (total)			Traumatic			Overload	
Variables	OR	(95% CI OR)	*P*	OR	(95% CI OR)	*P*	OR	(95% CI OR)	*P*
FM%	1.00	(0.92–1.09)	0.97	1.09	(0.97–1.23)	0.13	0.95	(0.84–1.07)	0.84
FM (kg)	1.00	(0.91–1.09)	0.91	1.06	(0.94–1.20)	0.30	0.95	(0.84–1.08)	0.50
BMC (kg)	1.04	(0.47–2.34)	0.91	1.21	(0.41–3.53)	0.71	0.88	(0.28–2.80)	0.84
FFM (kg)	1.00	(0.95–1.05)	0.85	0.97	(0.90–1.04)	0.46	1.01	(0.94–1.09)	0.63
BMD (g/cm^2^)	0.77	(0.03–19.2)	0.87	1.91	(0.02–154.6)	0.77	0.24	(0.00–22.3)	0.54
FM, upper limbs (g)	1.00	(1.00–1.00)	0.81	1.00	(1.00–1.00)	0.52	1.00	(0.99–1.00)	0.63
FM, lower limbs (g)	1.00	(1.00–1.00)	0.86	1.00	(1.00–1.00)	0.30	1.00	(1.00–1.00)	0.31
FM, trunk (g)	1.00	(1.00–1.00)	0.98	1.00	(1.00–1.00)	0.33	1.00	(1.00–1.00)	0.72
FFM, upper limbs (g)	1.00	(1.00–1.00)	0.85	1.00	(1.00–1.00)	0.85	1.00	(1.00–1.00)	0.52
FFM, lower limbs (g)	1.00	(1.00–1.00)	0.75	1.00	(1.00–1.00)	0.50	1.00	(1.00–1.00)	0.77
FFM, trunk (g)	1.00	(1.00–1.00)	0.90	1.00	(1.00–1.00)	0.37	1.00	(1.00–1.00)	0.52
BMC, upper limbs (g)	1.00	(0.99–1.01)	0.94	0.99	(0.99–1.00)	0.87	1.00	(1.00–1.00)	0.87
BMC, lower limbs (g)	1.00	(1.00–1.00)	0.85	1.00	(1.00–1.00)	0.64	1.00	(1.00–1.00)	0.79
BMC, trunk (g)	1.00	(1.00–1.00)	0.82	1.00	(1.00–1.00)	0.75	1.00	(1.00–1.00)	0.88

*Abbreviations: OR* odds ratio, *95% CI* 95% confidence interval, *%FM* relative fat mass, *FM* fat mass, *BMC* bone mineral content, *FFM* fat-free mass, *BMD* bone mineral density, *NS* non-significant

Finally, Table 4 presents the logistic regression results comparing body composition variables between the non-injured group and the group with injury in the lower limbs and between the non-injured group and the group with injury in the upper limbs. Body composition variables were not found to be risk factors.

**Table 4:** Logistic regression comparing the non-injured group and groups with lower or upper limb injury

Non-injured group vs. group with lower limb injury	B	SE	*P*	OR	(95% CI OR)	
					Lower	Upper
Bone mass, left lower limb	−0.002	0.002	0.468	0.998	0.994	1.003
Bone mass, right lower limb	−0.002	0.002	0.403	0.998	0.993	1.003
Difference between the lower limbs	−0.005	0.011	0.673	0.995	0.975	1.017
Fat mass, left lower limb	0.000	0.000	0.641	1.000	0.999	1.000
Fat mass, right lower limb	0.000	0.000	0.599	1.000	0.999	1.000
Difference between the lower limbs	−0.001	0.002	0.701	0.999	0.996	1.003
Lean mass, left lower limb	0.000	0.000	0.394	1.000	1.000	1.000
Lean mass, right lower limb	0.000	0.000	0.390	1.000	1.000	1.000
Difference between the lower limbs	0.000	0.001	0.946	1.000	0.999	1.001
Non-injured group vs. group with upper limb injury	B	SE	*P*	OR	Lower	Upper
Bone mass, left upper limb	0.005	0.009	0.607	1.005	0.987	1.023
Bone mass, right upper limb	0.005	0.009	0.576	1.005	0.988	1.023
Difference between the upper limbs	0.008	0.033	0.801	1.008	0.944	1.077
Fat mass, left upper limb	−0.001	0.002	0.708	0.999	0.996	1.003
Fat mass, right upper limb	−0.001	0.002	0.602	0.999	0.995	1.003
Difference between the upper limbs	−0.002	0.005	0.723	0.998	0.990	1.007
Lean mass, left upper limb	0.000	0.001	0.985	1.000	0.999	1.001
Lean mass, right upper limb	0.000	0.001	0.991	1.000	0.999	1.001
Difference between the upper limbs	0.000	0.002	0.979	1.000	0.996	1.004

*Abbreviations: B* beta coefficient of logistic regression, *SE* standard error, *OR* odds ratio, *95% CI* 95% confidence interval

## Discussion

Our prospective study with a follow-up period of 9 months investigated the prevalence of MI in Brazilian military students. We also sought to identify the risk factors for MI, overload and traumatic injuries, and upper and lower limb injuries. Of the participants, 27.3% presented with at least one type of MI, and overload injuries were the most prevalent (62.1%), with most injuries occurring in the lower limbs (69.7%). However, none of the studied body composition variables were found to be risk factors for MI, overload and traumatic injuries, or injuries in the lower or upper limbs. Finally, sports experience prior to military service was identified as a protective factor for MI.

The prevalence rate of almost 30% for MI in our study population reflects that MI is an important public health problem that deserves attention from military health-care providers, which has already been described by several studies. For example, an Iranian military study with a follow-up period of 1 year observed that MI accounted for 96% of health problems occurrence in one year of follow-up [1]. Moreover, a Finnish study demonstrated that 10% of a military sample was prematurely discharged from military service for medical reasons, mainly MI, and that premature discharge from military service was a potential risk factor for psychological problems, primarily in countries where military service is compulsory [22]. The findings of previous studies in the literature highlight the need for preventive strategies based on scientific knowledge about risk factors, considering the physical demands imposed on militaries in service that increase their risk for injury. Although we do not have epidemiologic data on other health problems in our study population, the incidence of MI in our study and previous epidemiologic studies in the literature can evidently justify the search for risk factors for MI.

With respect to the anatomical body parts affected, similar to our study, many studies have found a higher prevalence of injuries in the lower limbs. A Finnish study indicated a prevalence rate of 67% for lower limb injury in four cohorts followed up for 6 months [4], whereas another Finnish study reported a prevalence rate of 48% for lower limb injury in 944 conscripts who were followed up [3]. These results are consistent with those of our study, which showed that 69.7% of the injuries occurred in the lower limbs.

An interesting finding of our study is that sports experience prior to military service was a protective factor for MI, which is consistent with that of another military study [4]. According to Taanila et al. [4], previous experience of physical activity can produce overload on musculoskeletal structures prior to military service. In this case, we believe that previous physical activity programs can improve fitness and maturation of the musculoskeletal system. This could supposedly prepare participants for new training routines in the service, as the physical demands on them are lower than their less active counterparts. Such an idea was already previously proposed in the literature [23]. Moreover, this becomes evident in the study by Knapik et al. (2006) in which military low-fit recruits who participated in a pre-conditioning physical program before basic combat training tended to have a lower risk of injury during military service than low-fit recruits who did not participate in a pre-conditioning program [24].

This is particularly important for our study population, which was composed of students who supposedly had to spend part of their time studying for intelligence tests prior to military service. It is important to mention that, different from recruits, the military service as student in Escola Preparatória de Cadetes do Exército is not compulsory, and that before been considered approved to the service, participants underwent to a selection process composed by physical, health and intelligence test to be eligible.

However, it is important to mention that our main objective was to verify if anthropometric and body composition variables could be considered risk factors for MI. Despite our previous hypothesis, we could not identify any studied variables that could be considered risk factors for MI or overload or traumatic injuries. In this respect, no consensus on the relationship between body composition and anthropometric variables and the risk for MI exists in the literature.

Our hypothesis that anthropometric and body composition variables could be risk factors for MI was based on various previous studies. For example, in the military and athletic populations, higher BMI [3, 25], larger abdominal girth [3], and both low [26] and high body mass [27] were identified as risk factors for MI. Moreover, a high BMI was identified as a risk factor for MI in the lower limbs [28] and increased height was identified as a risk factor for MI [29]. Taking all these findings from previous studies into consideration, we formulated the hypothesis that body composition variables could also be risk factors for MI in our study population.

In contrast, although a small body of literature provides information on anthropometric variables as risk factors for MI, many studies that had the same objective refuted this hypothesis. For example, Rauh et al. observed that body mass, height, and BMI were not associated with incidence and risk of stress fractures or overload injuries in female recruits from the American Navy [30]. Moreover, some studies on athletes were not able to identify anthropometric variables as risk factors. For example, body mass was not considered a risk factor for MI in rugby athletes [31], and body mass, height, BMI, and body fat percentage measured by skinfold thickness were not identified as risk factors for MI in football athletes [32]. Consequently, the variability and inconsistency in results to date affect whether researchers can identify anthropometric variables as risk factors for MI.

Moreover, some studies sought to identify anthropometric variables as risk factors for specific injuries in militaries. Rauh et al. did not identify body mass, height, or BMI as a predictive factor for stress fractures [30] and Mahieu et al. did not also consider these variables as risk factors for calcaneus tendinopathy [33]. Further, Moen et al. did not identify body mass, height, BMI, maximal calf girth, and lean calf girth (maximal calf girth less calf skinfold) as risk factors for medial tibial stress syndrome [34].

Some hypotheses may potentially explain why our study, unlike other studies on militaries [3, 25] did not find a relation between anthropometric and body composition variables and the risk for MI. Our study population comprised students who were recently approved in a selection process prior to military service, which consisted of an intelligence test, followed by health examination and physical fitness tests. The characteristics of our study population may have reduced the variability in body composition variables in the students who were the sample population because of the physical fitness requirements to be approved in the selection process, which likely made our study population quite homogenous compared to the recruit population followed up in other studies. A study identified higher BMI as a risk factor for MI in American male recruits [35]. However, the mean BMI of the population in the previous study was 24.3 kg/m^2^, whereas the mean BMI of our study population was 22.9 kg/m^2^. Moreover, the standard deviation for BMI was 4.85 and 2.1 in the previous study and our study, respectively. It is also important to mention that no minimal physical fitness requirements for the recruits at the beginning of compulsory military service usually exist in Brazil; in contrast, the students in our study voluntarily participated in the military selection process, which had physical requirements for approval.

With respect to tools used to evaluate anthropometric and body composition variables in the previous studies, most investigations had measured anthropometric variables using simple and unspecific tools, and studies often failed to evaluate body composition variables or distinguish lean mass, fat mass, or bone variables. No military studies apparently used DXA to measure body composition variables to identify risk factors for MI in militaries. All anthropometric variables evaluated by the different aforementioned studies to date included body mass, height, BMI, abdominal girth, body fat percentage measured by skinfold thickness, maximal calf girth, and lean calf girth. In this regard, some disadvantages of DXA may explain why there are few studies, even with athletes, with the same objective as our study, that have investigated similar variables by using DXA: it is expensive, often not accessible to study centres, and is not portable.

A careful literature search revealed that a few studies indeed have used DXA with the same objective as the present study [36, 37] and suggests that many other body composition variables should be further investigated. In this respect, we found three studies that followed up athletes. Interestingly, of these three studies, only one identified body composition variables as risk factors. In this study, lower BMD, lower lean mass, lower bone (tibial) mass, higher fat mass, and higher body mass were associated with bone injuries [19]. The other two studies were not able to identify risk factors for stress fractures in cross-country athletes [36], and vertebral fractures in rugby athletes [37].

DXA continues to be considered the gold standard for evaluating BMD, being a non-invasive method that involves low radiation exposure, and demonstrates good accuracy with respect to total or segmental body composition evaluation [38].

However, aside from the advantages of the method, the fact that we could not find studies that used DXA to identify risk factors for MI in the militaries clearly indicates the current need for more studies adopting DXA with the same objective, preferably with a large sample of injured participants.

The small sample of injured participants can be considered a limitation of the present study, considering that there are previous studies with larger samples of significantly injured participants [3, 4], which gives them a higher statistical power for observing risk factors.

Furthermore, it would be interesting to register the severity of injuries as training days lost per injury, which would permit statistical analyses in groups categorized by severity. Finally, the cross-sectional design of body composition evaluation did not permit us to identify how body composition changed during military training. Thus, a no one-time point future study would be interesting.

However, considering the several strong points of this study and its results, and the evident variability of results found in the literature to date, this indicates the need for the identification of other intrinsic and extrinsic variables as risk factors. Our findings cannot confirm the relation between anthropometric and body composition variables and the risk for MI during military service.

## Conclusion

MI is an important public health problem that causes premature discharge from military service; 30% of our study population presented with MI. Lower limb injuries were the most prevalent, mainly ankle sprain and medial tibial stress syndrome. Overload injuries were more prevalent than traumatic injuries. It was not possible to establish the relationship between anthropometric or body composition variables and risk for MI during military service in this population with the current sample size. However, future studies with data collected over multiple time-points or with more individuals may identify patterns of injury risk.

## Abbreviations

BMC: Bone mineral content
BMD: Bone mineral density
BMI: Body mass index
DXA: Dual-energy X-ray absorptiometry
MI: Musculoskeletal injuries
